# Dyssegmental dysplasia, Silverman‐Handmaker type: A challenging antenatal diagnosis in a dizygotic twin pregnancy

**DOI:** 10.1002/mgg3.379

**Published:** 2018-03-11

**Authors:** Shuaa Basalom, Yannis Trakadis, Roberta Shear, Michel E. Azouz, Isabelle De Bie

**Affiliations:** ^1^ Department of Medical Genetics McGill University Health Centre Montréal QC Canada; ^2^ Department of Obstetrics and Gynecology Jewish General Hospital Montréal QC Canada; ^3^ Department of Radiology McGill University Health Centre Montréal QC Canada

**Keywords:** dyssegmental dysplasia, molecular diagnosis, prenatal diagnosis, skeletal dysplasia

## Abstract

**Background:**

Dyssegmental dysplasia Silverman‐Handmaker (DDSH; MIM 224410) type is an extremely rare skeletal dysplasia caused by functional null mutations in the perlecan gene. Less than forty cases are reported in the literature, of which only four were prenatally detected.

**Methods:**

We report on a dizygotic twin pregnancy from consanguineous parents for which one of the twins presented prenatally with severe micromelia, limb bowing and scoliosis, and postnatally with clinical and radiological features compatible with a diagnosis of dyssegmental dysplasia. Molecular studies were undertaken to confirm the clinical diagnosis of DDSH.

**Results:**

Molecular analysis results revealed a novel homozygous variant in the *HSPG2* gene (MIM 142461), NM_005529.6(*HSPG2*):c.4029 + 1G>A, consistent with a diagnosis of DDSH.

**Conclusion:**

To the best of our knowledge, the current report is only the seventh molecularly confirmed case of DDSH.

## 1 INTRODUCTION

Dyssegmental dysplasia (DD), also known as anisospondylitic camptomicromelic dwarfism or dyssegmental dwarfism includes two clinical subtypes, part of a clinical spectrum: Silverman‐Handmaker type (DDSH; MIM 224410) and Rolland‐Desbuquois type (DDRD; MIM 224400). DD is a rare autosomal recessive condition with a reported incidence of less than 1 in 1,000,000 (Spranger, Brill, Nishimura, Superti‐Furga, & Unger, 2012).^ ^DDSH is considered the severe clinical form, with high associated lethality. To date, only six cases of molecularly confirmed DDSH and 38 cases of DDRD have been reported in the literature (Ladhani et al., 2013; Rieubland et al., 2010).

Clinical features of DDHS include a flat midface, narrow thorax, abnormal ears, short neck, severe short stature, short and bowed limbs, as well as decreased joint mobility. DDSH patients also often present with cleft palate and club feet. The vertebral bodies of DDSH patients have patchy, multiple and irregular ossification centers defects, hence the term DD (Arikawa‐Hirasawa, Wilcox, & Yamada, 2001). The majority of neonates reported to be affected with DDSH die in the neonatal period.

## 2 CASE REPORT

Ethical compliance: ethics committee approval for case reports that do not include patients' photographs is not required from our institution.

**Figure 1:**
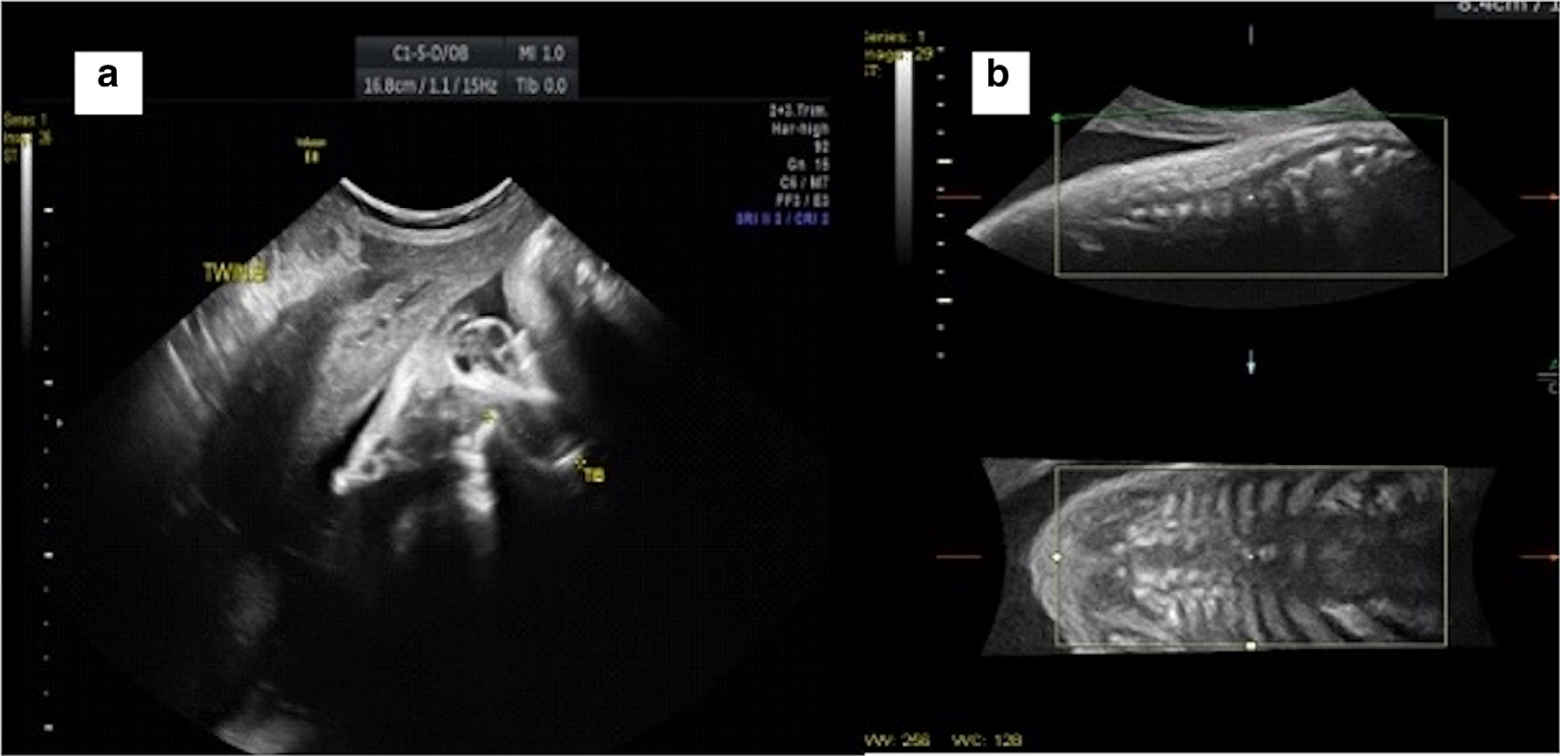
Fetal ultrasound (36 3/7 weeks): A. extreme bowing of tibia and B. vertebral segmentation defects
ο Marked scoliosis with segmentation defects (Figure 1B), a small thorax and relative cardiomegaly
ο Bone density was reported as normal, there were no detectable fractures

Our patient was the product of a spontaneous dizygotic twin conception for a healthy first cousin couple of Pakistani descent. Family history was non‐contributory. First antenatal ultrasound performed in Pakistan at 15 weeks showed a very short femur in Twin 2 (male). Second trimester fetal anatomic survey raised the clinical suspicion of a severe skeletal dysplasia, with micromelia, bowing of femur and tibia, severe scoliosis and small thorax circumference**.** The mother subsequently moved to Canada. Detailed fetal anatomic survey performed at 33 weeks' gestation showed the following features:

• Normal fetal anatomy and growth for twin 1 (female)
• For twin 2: 
ο very short long bones with humeri, radii, tibiae, and fibulae all below the 5^th^ percentile
ο Marked curvature/angulation of the left femur (Figure 1A), as well as hypoplastic lungs

The mother was counseled regarding the likely poor prognosis, but given the late gestational age and to minimize risks to the other twin, opted not to undergo invasive testing or selective termination.

The twins were born at 38 weeks by spontaneous vaginal delivery. Twin 1 was a healthy girl and twin 2 was a limp male with spontaneous breathing efforts. He was admitted to the neonatal intensive care unit for respiratory support. Growth parameters were: head circumference of 32.5 cm (3rd‐15th percentile), length of 41 cm (<0.1th percentile) and weight of 2,300 grams (0.1‐3rd percentile).

He presented with the following features: large anterior fontanel, wide cranial sutures, mild midface hypoplasia, depressed nasal bridge, small mouth, low set ears, micrognathia, short neck, short spine, narrow chest, kyphoscoliosis, micromelia and bowing of all long bones, as well as bilateral talipes.

Skeletal survey confirmed anisospondyly, coronal clefts and lack of ossification of several vertebrae, sagittal cleft in the vertebral bodies of the cervical and lumbar spine, short, flared and horizontal ribs, short, broad tubular bones with bulbous ends, bowing and dumbell appearance of femora, tibiae and humeri, small sacral sciatic notches with broad pubic bones and ischia, consistent with a clinical diagnosis of dyssegmental dysplasia. The patient's neonatal course was complicated by severe respiratory issues. He passed away at the age of 2 months secondary to respiratory failure.

Molecular analysis revealed a novel homozygous missense variant in the *HSPG2* gene (MIM 142461) (NM_005529.6(*HSPG2*):c.4029 + 1G>A) in intron 32. His mother was subsequently found to be a heterozygous carrier of this same variant. Paternal testing could not be performed as the father was in Pakistan.

## 3 DISCUSSION

DDSH is an extremely rare autosomal recessive skeletal dysplasia. To the best of our knowledge, the current report is only the seventh molecularly confirmed case of DDSH. Prenatal diagnosis of these less common fetal skeletal dysplasias is challenging, because of the rarity of these conditions, their overlapping features, and phenotypic variability. Most antenatal features are only detected in the second trimester. Severe and usually lethal forms can sometimes be detected in the first trimester. The earliest prenatally detected case of DDSH was at 13 weeks gestation, based on a familial recurrence (Ladhani et al., 2013).

Other conditions with similar prenatal presentation include the milder form of Dyssegmental Dysplasia Rolland‐Desbuquois type (MIM 224400) as well as Kniest Dysplasia (MIM 156550). The radiological findings in Kniest dysplasia are similar but less severe, without anisospondyly and with less bowing of the tubular bones. Kniest dysplasia is a form of type‐II collagenopathy, usually sporadic.

DDSH is typically caused by functional null mutations in the gene *HSPG2* (Kniffin & McKusick, 1991), encoding the heparan sulfate proteoglycan 2 protein perlecan, a co‐receptor for basic fibroblast growth factor. Perlecan is a major component of basement membranes, and is present in many tissues, including cartilage. *HSPG2 *is a large gene encompassing 97 exons, which maps to chromosome 1p36.1 (Arikawa‐Hirasawa et al., 2001; Kniffin & McKusick, 1991).

Perlecan is a large protein, functionally subdivided into five domains. Exons 13 – 40 encode domain III (Bhowmick et al., 2016). Domain III modulates the efflux of perlecan into extracellular space. An alteration in this domain is therefore expected to impair the proper localization of perlecan (Gubbiotti, Neill, & Iozzo, 2017).

The exact impact of the intron 32 splice variant identified in our patient is unknown, as no functional studies were performed. It was assumed, based on its location at an invariant splice site, that it would result in the formation of an aberrant mRNA and ultimately in lack of perlecan secretion. Given clinical features, sequence location, homozygous status in our patient and heterozygous carrier status in his mother, the variant was presumed causative. This particular variant has a reported total population allele frequency of 6.832e−05 in the ExAC database, and a slightly higher allele frequency (0.0003169) in the South Asian population (http://exac.broadinstitute.org/variant/1-22199112-C-T).

While Dyssegmental dysplasia Rolland Desbuquois type is considered the milder spectrum of DD, it is unclear at this time if this condition is related to *HSPG2*, as mutations in *HSPG2* have not been reported so far in DDRD patients (McKusick, 1986). Aside from DDSH, Schwartz‐Jampel Syndrome type 1 (MIM 255800) is also a perlecanopathy. This disorder is characterized by short stature, osteochondrodysplasia, myotonia, and a characteristic facies with “fixed” facial expression, blepharophimosis, pursed lips, and, sometimes, low‐set ears and myopia. Skeletal abnormalities include kyphoscoliosis, platyspondyly with vertebral coronal clefts, metaphyseal and epiphyseal dysplasia, and joint contractures. Unlike DDSH which results from null mutations in *HSPG2*, mutations associated with SJS are hypomorphic, loss‐of‐function variations which result in reduced levels of the perlecan protein, that is secreted to the extracellular matrix and likely partially functional (Arikawa‐Hirasawa et al., 2002; Stum et al., 2006).

Prabhu et al. reported the longest survival case of clinically diagnosed DDHS at 8 month of age. They attributed this prolonged survival to advances in medical care, possible gene modifiers and an intermediate phenotype (Prabhu, Kozma, Leftridge, Helmbrecht, & France, 1998). Another prolonged survival at four months for a patient with a clinical diagnosis of DDSH was reported by Winship et al. (Winship & Beighton, 2008), while the vast majority of other patients with DDHS have died in the immediate perinatal period. The cases reported by Prabhu and Winship were diagnosed on the basis of clinical findings, without molecular confirmation. See Table 1 for a comparison of reported DDSH cases.

**Table 1:** Clinical features and variants in molecularly confirmed cases of DDSH

Case	*HSPG2* variant a	Clinical features	Pregnancy outcome	Author
1	89‐bp dup homozygous (E 34)	Birth length less than second centile. Flat face, wide nasal bridge, posteriorly rotated ears, small mouth, micrognathia. Small chest, micromelia, anisospondyly and short, bent long bones. Other: pulmonary hypoplasia	Born at 39 weeks. Deceased shortly after	Arikawa‐Hirasawa et al., 2001;
2	89‐bp dup homozygous (E 34)	Clinical features similar to those of case 1.	T at 21 weeks	
3	G7086 + 5A (I 52); C10328T (E 73) compound heterozygous	Clinical features similar to those of case 1. Other: microcephaly, pterygium, exophthalmos, bilateral cataract, and posterior encephalocele.	T at 22 weeks	
4	4‐bp del homozygous (E 31)	Severe micromelia, narrow thorax, short ribs, short and bent long bones, anisospondyly of two vertebral bodies. Other: Encephalocele.	T at 15 weeks	Rieubland et al., 2010;
5	c.646G>T (E 7); c.5788C>T (E 46) compound heterozygous	Multiple vertebral segmentation abnormalities, poorly ossified vertebral bodies irregular in size and in shape, markedly shortened bowed long bones with dumbbell configuration, short iliac bodies with narrow notches, small chest, ribs horizontally oriented, clubbed feet. Short forehead, high arched palate, tethered tongue, micrognathia	T at 23 weeks	Ladhani et al., 2013
6	c.1356‐27_1507 + 59 del homozygous (E and I 12)	Severe micromelia with broad metaphyses, spine disorganization, small thorax. Flat face with severe micrognathia Other: joint contractures.	T at 13 weeks	
7	NM_005529.6(*HSPG2*):c.4029 + 1G>A homozygous (I 32)	Narrow chest, short, flared and horizontal ribs, short spine, kyphoscoliosis, anisospondyly, coronal clefts and lack of ossification of several vertebrae, sagittal clefts in the vertebral bodies of the cervical and lumbar spine, micromelia, short, broad tubular bones with bulbous ends, bowing and dumbell appearance of femora, tibiae and humeri, small sacral sciatic notches with broad pubic bones and ischia, bilateral club feet, mild midface hypoplasia, micrognathia. Other: large anterior fontanel, wide cranial sutures, depressed nasal bridge.	Born at 38 weeks; Deceased at 2 months	Present case

Hom (homozygous), bp (base pair), dup (duplication), del (deletion), E (exon), I (intron), PI (pulmonary insufficiency), T (termination of pregnancy).

a Variants nomenclature reported as cited.

A retrospective study by Schramm, which included 178 fetuses, reported on the difficult prenatal sonographic diagnosis of fetal skeletal dysplasia, given the rarity of these conditions and the large differential diagnosis. In this study, of the 160 fetuses with a confirmed diagnosis of skeletal dysplasia, a single case of DDRD case was antenatally diagnosed as “either osteogenesis imperfecta or campomelic dysplasia.” In a comparative retrospective study on the diagnostic accuracy of prenatal ultrasound for skeletal dysplasias, the highest reported accurate diagnostic yield was 68%. Although Ladhani et al. detected a case of DDSH as early as 13 weeks gestation (based on a familial recurrence), the twin conception and late gestational age in the current case provided additional challenges to the interpretation of sonographic features (Schramm et al., 2009). This article adds to the current knowledge on this rare skeletal dysplasia, particularly in the context of few molecularly confirmed cases.

Although DDSH and DDRD are considered part of a clinical continuum, there are at this time no molecularly confirmed cases of DDRD reported in the literature. This could be because the majority of cases were recounted before more widespread availability of molecular diagnosis. It is also possible that DDRD could result from variations in another gene within the perlecan pathway. An example of such genetic heterogeneity is found in the acromelic dysplasia group that includes Weill‐Marchesani syndrome (WMS; MIM 614819), Geleophysic Dysplasia (GD; MIM 231050) and Acromicric Dysplasia (AD; 102370). These three conditions present significant overlap of their clinical features. All can be associated with variant in the *FBN1* gene. Yet, cases of WMS have also been associated with variants in *LTBP2* and *ADAMTS10,* while GD is more commonly associated with variants in *ADAMTSL2* and *LTBP3* (Le Goff & Cormier‐Daire, 2009).

As for other conditions, interpretation of splice site or missense variants in *HSPG2* will remain challenging. Given that splice variants have been now reported in association with both Schwartz‐Jampel (the “milder” form of perlecanopathies) and DDHS (the lethal form of Dyssegmental Dysplasia [this case]), clinical prognosis, particularly of prenatally detected cases, will still heavily rely on clinical features. Systematic molecular and biochemical analysis of patients with suspected « perlecanopathies » would aid in gaining insight into the phenotype/genotype correlation of this complex molecule (Arikawa‐Hirasawa et al., 2001; Gubbiotti et al., 2017).

Molecular analysis for confirmation of the clinical diagnosis is important, but may not be sufficient to provide an accurate postnatal prognosis. However, availability of molecular testing provides the opportunity for pre‐implantation or prenatal testing in subsequent pregnancies.

Accession numbers and URLs for data in this article are as follows:

https://www.ncbi.nlm.nih.gov/nuccore/NM_005529.5/ for HSPG2 sequence cDNA [accession NM_005529]
https://www.omim.org/entry/142461?search=hspg2&highlight=hspg2
https://www.omim.org/entry/224410?search=224410&highlight=224410
https://www.omim.org/entry/224400?search=224400&highlight=224400
http://exac.broadinstitute.org/variant/1-22199112-C-T

## CONFLICT OF INTEREST

None declared.
